# Expression Analysis Reveals Differentially Expressed Genes in BPH and WBPH Associated with Resistance in Rice RILs Derived from a Cross between RP2068 and TN1

**DOI:** 10.3390/ijms241813982

**Published:** 2023-09-12

**Authors:** Rashi Anand, Dhanasekar Divya, Sudeshna Mazumdar-Leighton, Jagadish S. Bentur, Suresh Nair

**Affiliations:** 1 Plant-Insect Interaction Group, International Centre for Genetic Engineering and Biotechnology, New Delhi 110067, India; rashianand@icgeb.res.in; 2 Plant Biotic Interaction Lab, Department of Botany, University of Delhi, Delhi 110007, India; smazumdar@botany.du.ac.in; 3 Agri Biotech Foundation, Rajendranagar, Hyderabad 500030, India; divi41@gmail.com (D.D.); jbentur@yahoo.com (J.S.B.)

**Keywords:** *Nilaparvata lugens*, *Sogatella furcifera*, rice pests, gene expression analysis (GEA), recombinant inbred lines (RILs), integrated pest management (IPM), transcriptome, next-generation sequencing (NGS)

## Abstract

BPH (brown planthopper) and WBPH (white backed planthopper) are significant rice pests that often co-occur as sympatric species and cause substantial yield loss. Despite their genetic similarities, different host-resistance genes confer resistance against these two hoppers. The defense mechanisms in rice against these pests are complex, and the molecular processes regulating their responses remain largely unknown. This study used specific recombinant inbred lines (RILs) derived from a cross between rice varieties RP2068-18-3-5 (BPH- and WBPH-resistant) and TN1 (BPH- and WBPH-susceptible) to investigate the mechanisms of interaction between these planthoppers and their rice hosts. WBPH and BPH were allowed to feed on specific RILs, and RNA-Seq was carried out on WBPH insects. Transcriptome profiling and qRT-PCR results revealed differential expression of genes involved in detoxification, digestion, transportation, cuticle formation, splicing, and RNA processing. A higher expression of sugar transporters was observed in both hoppers feeding on rice with resistance against either hopper. This is the first comparative analysis of gene expressions in these insects fed on genetically similar hosts but with differential resistance to BPH and WBPH. These results complement our earlier findings on the differential gene expression of the same RILs (BPH- or WBPH-infested) utilized in this study. Moreover, identifying insect genes and pathways responsible for countering host defense would augment our understanding of BPH and WBPH interaction with their rice hosts and enable us to develop lasting strategies to control these significant pests.

## 1. Introduction

Plant–insect interaction is a complex evolutionary system where the arms race between two organisms constantly forces them to co-evolve multiple defense responses and develop strategies for their respective survival. When interacting, plants and insects exert and evolve in response to reciprocal natural selection, while co-diversification shapes the outcome of species interactions across various evolutionary scales. This concept has long been an appealing theoretical framework for developing viable pest management strategies. A large group of herbivores that comprises piercing and sucking insects target the plant phloem and use it as their primary source of nutrition. These insects, while feeding, secrete saliva into the plant tissues, which contain enzymes or effector molecules that interfere with the plant defense responses [1,2,3]. Several studies have reported plant resistance and defense responses to piercing and sucking insects, which include signal pathways that recognize the effectors present in insect saliva and trigger strong resistance reactions in host plants. Yet, our understanding of insect responses to resistant host plants is still limited, particularly at the genomic and transcriptomic levels.

Over the years, rice (*Oryza sativa*) and its insect pests have become critical model systems for understanding the intricacies of plant–insect interactions [4,5]. Moreover, rice is one of the most important food crops in the world today and suffers high yield loss due to its major insect pests. A significant sucking insect pest of rice is the brown planthopper (*Nilaparvata lugens*; BPH), followed by its close relative, the white backed planthopper (*Sogatella furcifera*; WBPH), which adversely affect rice yield and are also known vectors for plant viruses [6]. Both pests are highly migratory, capable of traversing long distances, and therefore invade almost all major rice-growing areas of the world. While BPH is monophagous, WBPH is an oligophagous insect that feeds on several other plants of the Poaceae family, apart from rice, including wheat and maize. Both species often co-occur and feed on the phloem sap of rice, sharing the same habitat throughout the rice growing season [7]. Several studies have shown mutual interspecific interactions between the two pests and their survival as sympatric species [7,8]. While there is considerable information on how plants interact with insect pests and the various strategies they adopt to overcome herbivore attacks, a good understanding of how insects interact and especially how BPH and WBPH deploy their defenses to overcome resistant rice varieties is scarce.

Conventional, biological, and chemical control, as well as microbial insecticide-based control strategies, have been commonly used for managing BPH and WBPH with varying levels of success. Though popular and extensively used in modern-day agriculture for pest management, chemical control methods are expensive and ecologically disruptive. Moreover, the indiscriminate use of a wide range of pesticides has led to insect resistance and pest resurgence, besides being potentially harmful to the environment. Consequently, this has affected food security worldwide [9,10]. Further, the development and time-to-time deployment of new insect-resistant rice varieties harboring new resistance genes against these pests has not provided long-term and durable solutions to combat these insects because of the latter’s ability to overcome host resistance rapidly. Therefore, a detailed understanding of the molecular strategies adopted by the insects to overcome host plant resistance will help design better and more durable management strategies against these pests. 

Recent multi-disciplinary studies focused on plant–insect/pathogen interactions have revealed several unconventional mechanisms relating to host resistance and susceptibility. In several cases, the resistance of plants to insects and pathogens is continuously evolving and controlled by quantitative trait loci (QTLs) [11,12]. Characterizing innate immunity and its interplay with quantitative resistance is critical for understanding the selective evolutionary pressures that shape quantitative resistance in plant hosts. In this context, several BPH and WBPH resistance genes have been identified and mapped in rice over the past few decades.

At least 43 major resistance genes and 72 QTLs conferring resistance to BPH and 19 major genes and 75 QTLs conferring resistance to WBPH are reported [1,13,14,15,16,17]. This information, coupled with the availability of linked molecular markers for many of the BPH and WBPH resistance genes, has led to a surge in molecular breeding in rice aimed at pyramiding R genes to provide durable resistance against BPH and WBPH. However, frequent breakdown of BPH/WBPH resistance in rice has also been reported [18,19]. The underlying mechanisms responsible could be a result or a combination of the emergence of new virulence genes or the modification of effectors in BPH [20,21], the dynamic nature of the endosymbionts present in these insects [22], or selective alterations in BPH metabolism [23].

In our earlier study [1], rice recombinant inbred lines (RILs) (F_14_) derived from a cross between the rice breeding line RP2068-18-3-5 (resistant to both BPH and WBPH) and TN1 (susceptible to both the planthoppers) were analyzed after insect infestation. This study revealed segregation of BPH and WBPH resistance among the RILs based on the overall performance of the insects, results from the seedbox screen test, and other phenotypic tests such as nymphal survival, nymphal preference, and days to wilt. RILs with 44–80% genetic similarity were shortlisted based on the four phenotypic tests, and molecular analysis revealed a large set of differentially expressed genes (DEGs) in these lines that participate in several pathways critical for rice defense against the two planthoppers. Further, evaluation against BPH and WBPH indicated that the resistance to the two hoppers is independent of each other in these lines and likely conferred by separate genes. Furthermore, this study also revealed differences in gene expression among the respective BPH- and WBPH-resistant RILs after exposure to BPH or WBPH. While the earlier study highlighted the plant responses to these hoppers at the molecular level, these factors warranted further investigation to obtain a deeper understanding of the responses of the two hoppers to the resistance genes in the respective RILs. 

In the current study, four RILs that were categorized as SS (susceptible to BPH and WBPH; TR24SS), RS (resistant to BPH and susceptible to WBPH; TR145RS), SR (susceptible to BPH and resistant to WBPH; TR152SR), and RR (resistant to BPH and WBPH; TR94RR) were used to elucidate the counter-defense mechanisms of the insects (BPH or WBPH) to these hosts with differential resistance against the two hoppers but almost identical genetic background. Furthermore, this information would also help us understand the corresponding survival strategies the insects adopt to overcome plant resistance.

Although the complexity of the defense mechanisms of these hoppers against host plant resistance is well studied, their underlying molecular mechanisms remain largely unknown. Our earlier study [1] showed that in the rice breeding line, RP2068-18-3-5 and RILs developed from this line harbored different host-resistance genes that confer resistance against these two planthoppers. WBPH and BPH were allowed to feed on specific RILs as stated earlier (i.e., SS, RR, RS, and SR lines) to explore and identify mechanisms of interactions between these planthoppers and their rice hosts. Subsequently, RNA-Seq analysis was carried out on these insects, followed by quantitative real-time PCR for selected genes to validate the results obtained through RNA-Seq. 

To the best of our knowledge, this is the first comparative analysis of differential gene expressions in these insects fed on genetically similar hosts that show differential resistance to BPH and WBPH. These results complement our earlier findings [1] on differential gene expression of the same RILs (after infestation with BPH or WBPH) utilized in the current study. Furthermore, while augmenting our understanding of the interaction of BPH and WBPH with their rice hosts, these results also provide valuable information for developing durable strategies to control and manage these significant rice pests.

## 2. Results

### 2.1. WBPH RNA-Seq and Differential Gene Expression (DGE) Analysis

**Figure 1:**
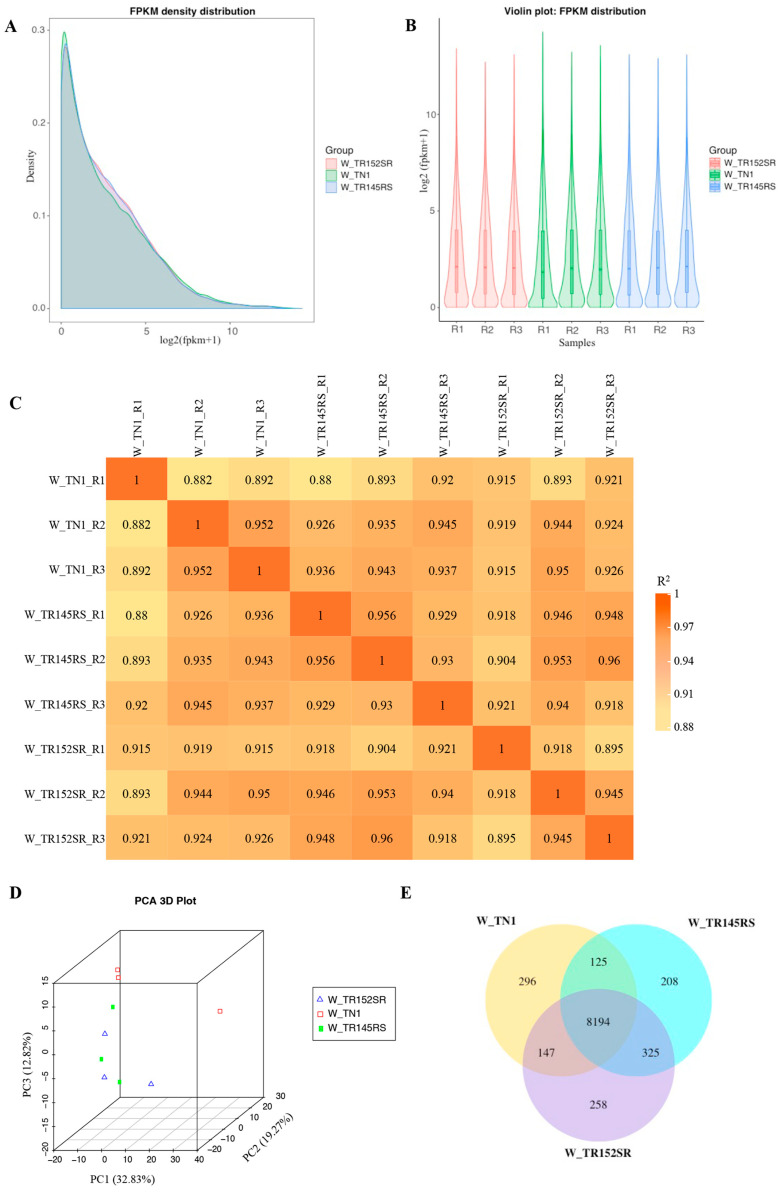
Gene expression level analysis in WBPH. (**A**) FPKM (fragments per kilobase of exon per million mapped fragments) density distribution plot. The density distributions depict 21,254 transcripts as a measure of gene expression levels in each sample. (**B**) Violin plot indicating distributions of gene expression data of nine samples post normalization. The *X*-axis represents the three replicates of each sample, and the *Y*-axis indicates log2 (FPKM+1) values. (**C**) Pearson correlation coefficient matrix, depicting the correlation of the gene expression levels between samples and indicating the reliability of the experiments. (**D**) Principal Component Analysis (PCA) plot showing the variance of the three biological replicates of each of the three samples (W_TN1, W_TR145RS, W_TR152SR). The figures on each axis represent the percentages of variation explained by the principal components. (**E**) Co-expression Venn diagram indicating common and uniquely expressing genes in the three samples. The prefix ‘W_’ refers to WBPH samples.

Nine WBPH libraries were constructed and sequenced (RNA-Seq), and the number of raw reads generated from each library ranged from 21.4 to 26.8 million and the clean reads ranged from 20.9 million to 26.3 million (Table 1). The Q20 and Q30 values for all the libraries were higher than 97% and 93%, respectively. The GC content of the clean reads of each sample was also calculated (Table 1). More than 86% of the clean reads were successfully mapped to the reference genome, of which 61–66% were uniquely mapped (Table S3). The FPKM values represented transcript abundance and directly reflected gene expression levels across all samples. Our data for all nine libraries revealed that FPKM values are similar, indicating comparable coverage across all the libraries. Further, the density distribution of mRNAs (Figure 1A) and the gene expression profiles of different samples are presented as a violin plot (Figure 1B). The R^2^ ranged from 0.88 to 0.969 between the different samples of the three groups (Figure 1C). Figure 1D shows the PCA plot depicting the possible correlated variation between the different samples of the three groups, W_TN1, W_TR145RS, and W_TR152SR. The number of genes uniquely expressed in WBPH within each group is shown in Figure 1E. The overlapping regions show the number of co-expressed genes in two or more groups. 

**Figure 2:**
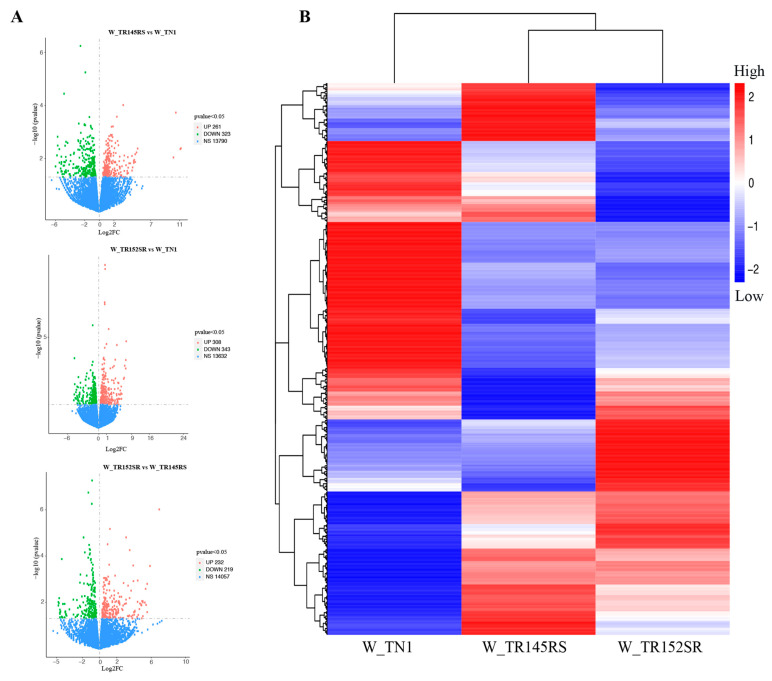
Differential gene expression analysis of WBPH feeding on the different rice lines. (**A**) Volcano plot depicting the overall distribution of differentially expressed genes in the three groups W_TR145RS vs. W_TN1, W_TR152SR vs. W_TN1, and W_TR145RS vs. W_TR152SR. The horizontal axis denotes the fold change, while the vertical axis denotes the statistically significant differences in gene expression levels in the different groups. The squares represent genes; red and green squares indicate upregulated and downregulated genes, respectively, and blue squares indicate changes in gene expression levels that are statistically non-significant. (**B**) Hierarchical clustering heatmap depicting overall results of the FPKM clustering using log2 (FPKM+1) values. The red and blue squares indicate genes with high or low gene expression levels, respectively.

In our study, the read count (FPKM) obtained from RNA-Seq was further processed and analyzed to better represent the differential gene expression in the samples (Table S4). In addition, the reliability of our data was enhanced because of the tight control over the false discovery rate (using the Benjamini and Hochberg [24] approach; see Materials and Methods section for details). Figure 2A indicates the number of genes differentially expressed between sample pairs, TN1 vs. RS, TN1 vs. SR, and RS vs. SR. A total number of 14,374 genes were identified to be differentially expressed when TN1 was compared to RS, out of which, 261 and 323 were significantly upregulated and downregulated, respectively (*p* < 0.05). In addition, 15 and 6 DEGs showed a more than five-fold decrease and increase in their expression levels, respectively. Similarly, when TN1 was compared to SR, out of a total of 14,283 DEGs, only 308 showed significant upregulation (*p* < 0.05), whereas 343 showed substantial downregulation; of these, 9 and 13 DEGs exhibited a more than five-fold decrease and increase in their expression levels, respectively. Also, when RS was compared to SR, 14,508 genes were shown to be differentially expressed; however, 232 and 219 genes were significantly up- and downregulated, respectively. It was observed that 17 DEGs showed a four-fold decrease in their expression, and 10 DEGs showed more than a five-fold increase in their expression levels. The overall results of the FPKM cluster analysis (Figure 2B) of more than 1000 differentially expressed genes were clustered using their log2 (FPKM+1) value in the form of a heat map (Supplementary Figure S1). Clustering genes with similar expression levels was carried out to better understand gene expression in relation to their biological relevance. Genes collated based on comparable expression levels indicated they likely have similar functions or participate in the same pathway or metabolic process.

### 2.2. GO and KEGG-Based Enrichment Analysis

**Figure 3:**
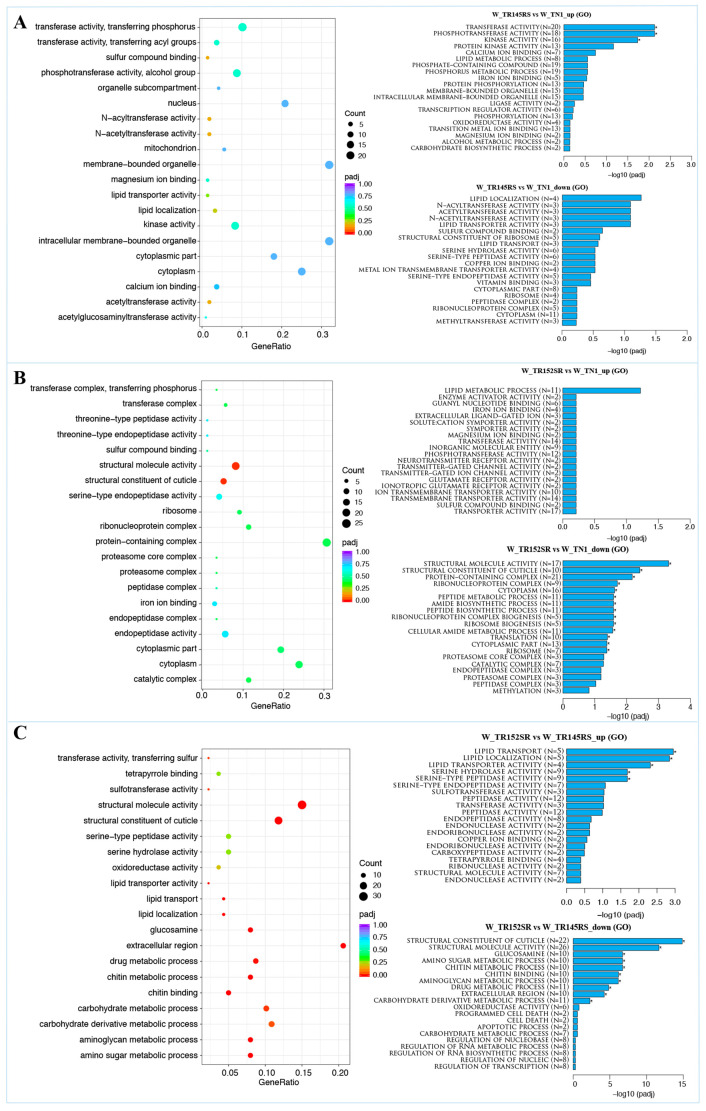
Gene Ontology enrichment analysis of the top 20 enriched GO terms in WBPH represented as dot plots. Differentially expressed genes were grouped into functional groups using ClusterProfiler (v3.8.1) software. The twenty most highly enriched GO terms, up- and down-regulated, are shown as bar plots. (**A**) GO enrichment analysis of Group1: W_TR145RS vs. W_TN1; (**B**) GO enrichment analysis of Group2: W_TR152SR vs. W_TN1; (**C**) GO enrichment analysis of Group3: W_TR152SR vs. W_TR145RS. Terms with a padj value < 0.05 were used as the standard for screening DEGs and were considered significantly enriched.

A deep analysis of the DEGs was conducted, including GO and KEGG enrichment analysis. GO enrichment analysis was performed to ascribe functions to DEGs and categorize them as biological processes, molecular functions, and cellular components. Figure 3 depicts the top 20 GO terms of the three groups: Group 1: WBPH insects fed on TN1 (control) with the WBPH fed on susceptible line (RS), Group 2: Control with WBPH fed on resistant line (SR), and Group 3: WBPH fed on susceptible (RS) with the resistant line (SR). Of the top 20 GO terms, 12 terms were classified under the molecular function category (mainly associated with the terms “transferase activity”, “kinase activity”, “sulfur compound binding”, and “magnesium ion binding”), seven under the cellular components category (mainly associated with terms “nucleus”, “mitochondria” “cytoplasm”, and “organellar components”), and only one under biological process (“lipid localization”) (Figure 3A). Amongst the top 20 GO terms, 11 were classified as cellular components (mainly associated with terms such as “protein-containing complex”, “enzymatic complex”, “cytoplasm” and “peptidase/endopeptidase complex”), and nine as molecular functions (“transferase activity”, “endopeptidase activity”, “sulfur compound binding” and “structural constituent of cuticle”) (Figure 3B). In addition, “transferase activity”, “transferring phosphorus-containing groups”, and “cytoplasm” were both downregulated when WBPH insects fed on susceptible and resistant lines were compared to the control (TN1). In contrast, “sulfur compound binding” was upregulated in the insect feeding on the resistant line and downregulated in the insect feeding on the susceptible line. Interestingly, when comparing gene expression in insects fed on susceptible and resistant lines, most terms fell either in the categories of biological processes (9) or molecular functions (11) (Figure 3C). Several metabolic processes were downregulated, whereas terms such as “serine-type peptidase activity”, “serine hydrolase activity”, and several “cell death/programmed cell death/apoptotic process” were upregulated.

**Figure 4:**
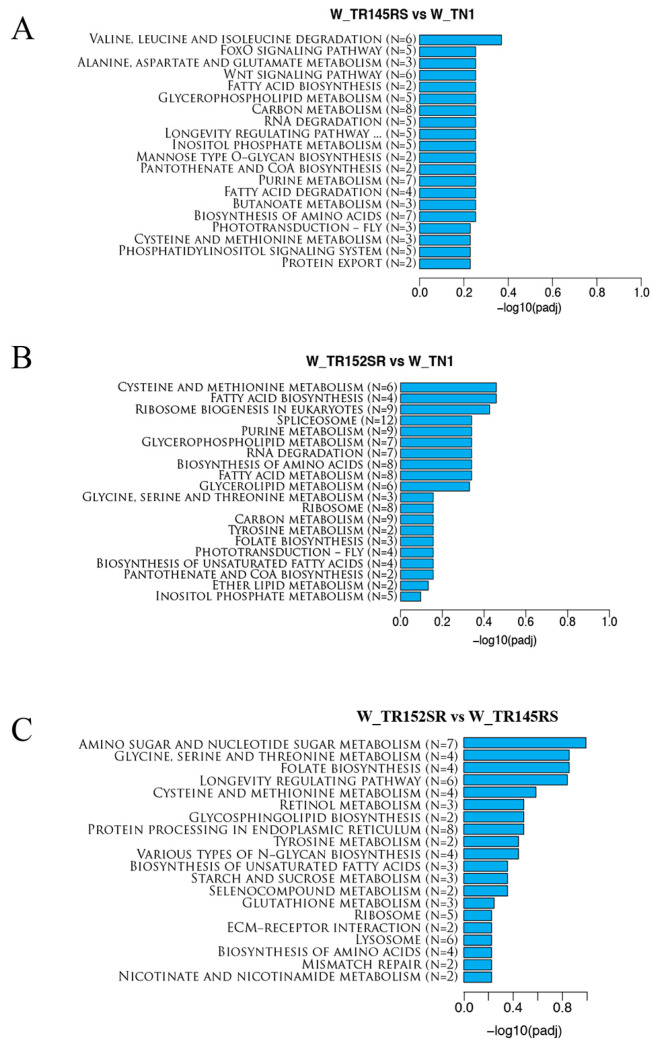
KEGG enrichment pathway analysis of the top 20 differentially expressed genes in WBPH represented as bar plots for the three groups, (**A**) W_TR145RS vs. W_TN1, (**B**) W_TR152SR vs. W_TN1, and (**C**) W_TR145RS vs. W_TR152SR. The differentially expressed genes were grouped into gene pathways using pathway enrichment analysis using the KEGG database and ClusterProfiler (v3.8.1) software.

A KEGG pathway-based analysis was performed using ClusterProfiler to further understand the biological functions and interactions of genes. The top 20 significantly enriched terms in the KEGG enrichment analysis are shown (Figure 4). A total of 82 KEGG-enriched pathways were observed in Group 1. The insects feeding on TN1 were compared to those feeding on RS (W_TR145SS vs. W_TN1), with 114 DEGs distributed in the enriched pathways. The 91 KEGG pathways were enriched with 150 DEGs in Group 2 (W_TR152SS vs. W_TN1). The 88 DEGs distributed in 68 KEGG-enriched pathways were observed in Group 3 (W_TR145SS vs. W_TR152SS). In Group 1, the terms enriched with the highest number of DEGs are related to carbon metabolism, purine metabolism, and biosynthesis of amino acids. In contrast, in Group 2, apart from carbon metabolism, other terms enriched with maximum DEGs are spliceosome, ribosome biogenesis in eukaryotes, and purine metabolism. In Group 3, the enriched terms with the highest number of DEGs were protein processing in the endoplasmic reticulum, longevity signaling pathway, and amino sugar and nucleotide sugar metabolism.

### 2.3. Comparative Expression Profiles of Select Genes of BPH and WBPH by qRT-PCR 

Based on earlier stated criteria (see Materials and Methods) and thresholds (log2 fold >2.5 and padj > 0.05), ten candidate genes (Table S2) were selected to validate their expression levels and also to compare gene expression profiles of WBPH insects fed on SS (TR24SS) and RR (TR94RR) rice lines. The RT-PCR results concurred with the results obtained through RNA-Seq expression data, indicating its reliability. 

**Figure 5:**
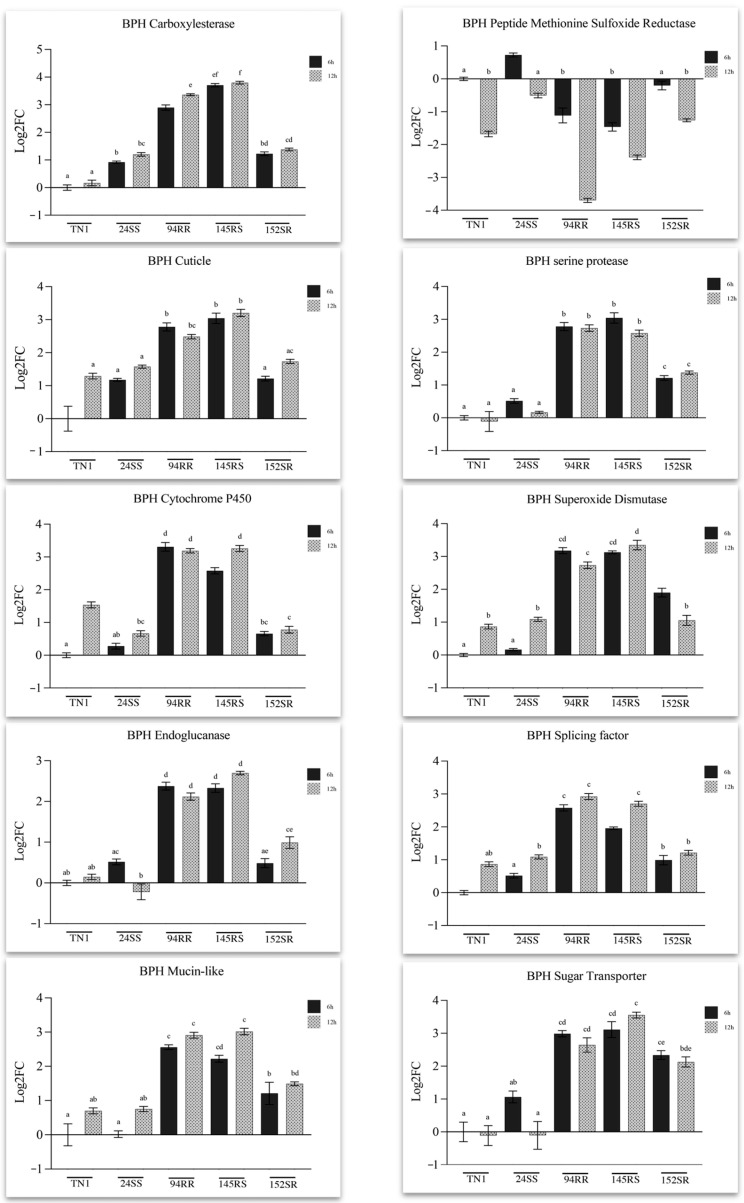
Relative expression (Log2FC) analysis of the ten shortlisted genes of BPH (see Materials and Methods for details) fed on 5 rice genotypes: TN1, TR24SS, TR94RR, TR145RS, and TR152SR at two different time points (6 h and 12 h after release). Error bars represent mean ± SE (*n* = 3). Bars with different letters are statistically significant (*p* < 0.05).

**Figure 6:**
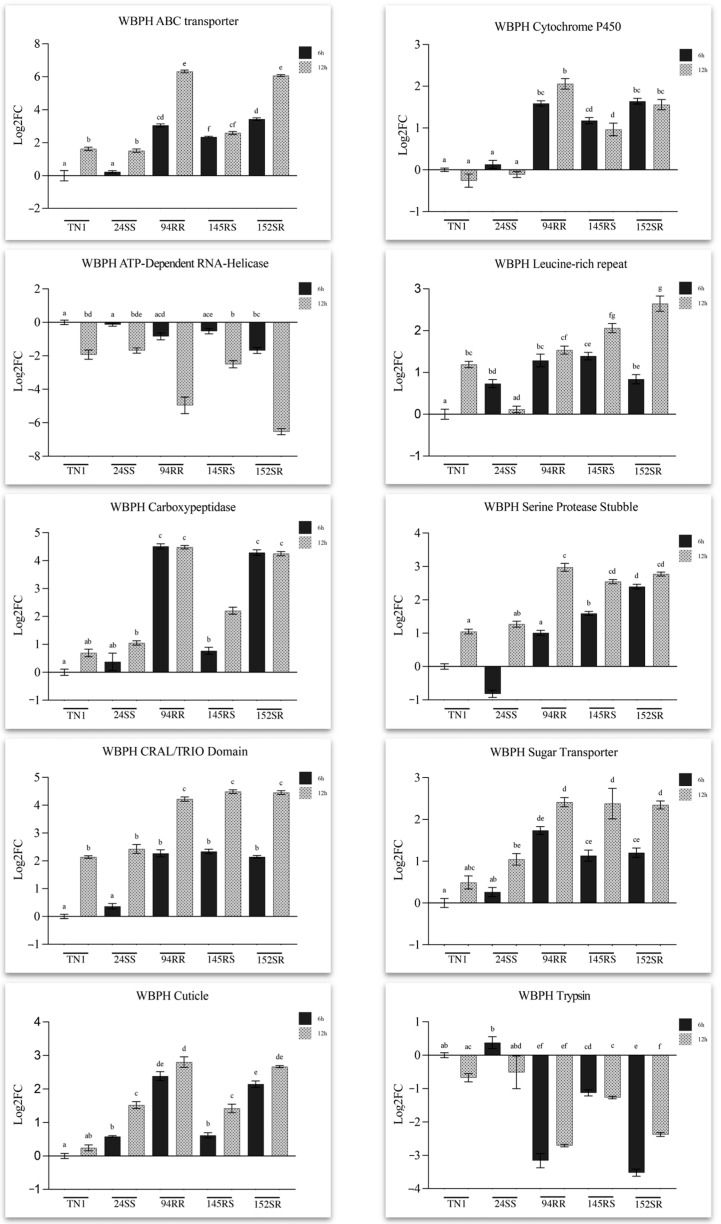
Relative expression (Log2FC) analysis of the ten shortlisted genes of WBPH (see Materials and Methods for details) fed on 5 rice genotypes: TN1, TR24SS, TR94RR, TR145RS, and TR152SR at two different time points (6 h and 12 h after release). Error bars represent mean ± SE (*n* = 3). Bars with different letters are statistically significant (*p* < 0.05).

The pattern of expression of four genes in the list of selected genes, i.e., cuticle protein, cytochrome P450, sugar transporter, and serine protease, was different in BPH (Figure 5) and WBPH (Figure 6). The gene expression pattern of cuticle protein, a structural gene, showed its induction early and sustained in both the insects when feeding on their respective resistant lines, BPH on the RR and RS lines and WBPH on the RR and SR lines. Cytochrome P450, a significant detoxification gene, displayed an early and sustained enhanced response in BPH fed on BPH-resistant lines (>3-fold). A basal induction of gene expression was also seen in BPH insects fed on TN1 samples (12 hai). In contrast, in WBPH, the change in expression levels of this gene was less than 2-fold in insects that fed on RR and SR lines. WBPH insects that fed on the susceptible RS line also showed higher expression of this gene than those insects feeding on TN1. In BPH, a serine protease gene responsible for digestion and other immune responses in most insect pests showed early and sustained induced expression levels in response to feeding on the resistant lines, and intermediary responses were observed when feeding on the line (SR) resistant to WBPH. In WBPH, another serine protease, a putative Trypsin, showed a negative response. This gene was downregulated (>3-fold) in WBPH insects that fed on the resistant host, and lower expression was observed in 6 hai samples compared to 12 hai samples. In BPH, a sugar transporter gene was upregulated when fed on resistant lines at both 6 and 12 hai. In addition, the insects fed on SR lines also showed higher levels of expression than those fed on TN1. In contrast, and interestingly, in WBPH, this gene showed a late response (gene expression induced at 12 h) against all three lines carrying resistance genes against one or both insects (RR, SR, and RS). 

Of the other BPH genes that were assayed by qRT-PCR (Figure 5), the carboxylesterase gene, reported to be involved in the detoxification of the plant defense molecules, was consistently induced (>3-fold) when feeding on the resistant lines. A similar response was also observed for the endoglucanase gene, another important insect defense gene reported to be involved in plant cell wall degradation and which acts as an effector that overcomes plant cell wall defense in rice, and mucin-like protein—a salivary protein crucial for insect feeding. Both these genes exhibited less than a 3-fold increase in expression when feeding on resistant lines compared to expression observed in insects feeding on the control plant (TN1). The peptide methionine sulfoxide reductase (PMSR) gene and superoxide dismutase (SOD) are two ROS (Reactive Oxygen Species) scavenging enzymes whose expression profiles varied drastically from each other in the current study. Though the SOD showed consistent upregulation in insects fed on the resistant lines, the PMSR gene showed a late downregulation response against the resistant lines. A more than 3-fold downregulation of this gene was observed in insects fed on the RR line at 12 hai. Another gene that is reported to play a critical role in the survival of the insects on resistant hosts is the serine/arginine-rich splicing factor. Though basal level induction was seen in all insects, a significant upregulation of this gene was observed in insects fed on RR and RS lines (Figure 5). 

Most of the WBPH genes whose expression was assayed through qRT-PCR showed a late response against the resistant lines (Figure 6). The ABC (ATP-binding cassette) transporter, a significant detoxification transporter involved in xenobiotic clearance, exhibited a late response in insects feeding on resistant hosts. At the 12 hai time point, more than a 5-fold change in the expression levels of this gene was seen in insects feeding on resistant RILs. However, a 2-fold induction was also seen in the WBPH when fed on the susceptible line (RS) compared to insects that fed on TN1 control plants. The ATP-dependent RNA helicase gene, critical for all aspects of RNA metabolism, was observed to be downregulated when fed on resistant hosts. Further, more than 4-fold decrease in expression levels was seen in insects fed on RR and SR lines at 12 hai time points. The CRAL/TRIO domain is a structural protein domain that has been reported to bind to a diverse set of small lipophilic molecules. The genes of the CRAL/TRIO domain showed late but significant upregulation in all insects fed on three resistant rice lines. Even though the RS line is susceptible to WBPH, expression of the CRAL/TRIO domain was similar in insects feeding on the other two resistant lines. In addition, at 12 hai, at least a 2-fold upregulation was observed in insects that fed on TN1 and SS lines. Carboxypeptidase, another dominant midgut digestive enzyme, showed an early and sustained response in WBPH feeding on the resistant hosts, and in contrast, very low expression levels were observed in the insects that fed on susceptible lines. The serine protease homolog, Stubble, which lacks the enzyme’s catalytic chain, is primarily involved in epithelial and cytoskeleton morphogenesis. This gene showed moderate to high upregulation in WBPH feeding on all three resistant lines. A higher response was observed in insects at the 6 hai time point, followed by a decrease in expression at 12 hai in insects feeding on the RR line. Leucine-rich immunoglobin domain genes showed inconsistent expression in insects fed on all the lines. 

## 3. Discussion

The co-evolutionary arms race between rice and its pests has led to an increasing diversity of plant defense mechanisms along with concomitant variation in insect adaptation and diversification. In the case of plant–insect interaction, the ‘escape and radiate’ model of coevolution has been invoked to explain the patterns of evolutionary diversification in the pest and the plant [25,26]. This process includes the evolution of the defense mechanisms in response to the pest-induced selective pressures, leading to adaptive radiation of the host. However, there remains a significant challenge in establishing links between micro-evolutionary processes and understanding the mechanisms involved in biological diversification patterns for both host plants and their insect pests.

During its evolutionary history, rice has developed several defense mechanisms against BPH, WBPH, and several other invaders [10,27]. Resistance to BPH is complex as it involves multiple sophisticated regulatory mechanisms both at the level of the insect and its host [28]. To protect themselves from pest attacks, plants develop constitutive and induced defense mechanisms [29,30,31,32]. While some defenses are the constitutive defense mechanisms that plants express regardless of the presence or absence of the pest, others are induced defense mechanisms that are triggered post-pest infestations, starting with recognizing specific insect-associated molecular patterns and, in the process, activating complex signaling networks [30,31,32,33]. A molecular approach for investigating the intricacies of plant–insect interactions is to comprehensively characterize the transcriptomes of the interacting partners during the course of the interaction to generate data-rich outputs. Subsequent analyses of the data can reveal valuable information on the interactions at the molecular level. The current study adds new insights into rice–WBPH interactions. Additionally, it highlights some differences in the response of the two pests, BPH and WBPH, when fed on genetically similar but differentially resistant hosts.

To identify differences and commonalities in the defense pathways invoked in rice against the two planthoppers, BPH and WBPH, results from our earlier study [1] identified various pathways that were triggered or suppressed in rice genotypes when challenged with BPH or WBPH. Transcriptome analyses of infested rice genotypes (RILs generated from a cross between TN1 × RP2068) indicated that resistance to the two planthoppers is independent. However, this study provided information only about the host when exposed to these two planthoppers. Therefore, to observe and understand the intricacies of this interaction, a critical understanding of the defense mechanism and molecular responses of the insect partners is equally vital. This study is the first comparative analysis of differential gene expressions in these insects fed on genetically similar hosts that show differential resistance to BPH and WBPH. Further, this complements our earlier findings [1], as the same insect material and RILs (BPH or WBPH infested) used in that analysis on the differential gene expression of rice hosts were utilized in the current study as well.

Employing omics tools, we investigated molecular defense pathways triggered or suppressed in BPH and WBPH when feeding on rice lines resistant to BPH and/or WBPH in relation to those feeding on susceptible lines (see Section 4 for details). The RNA-Seq data analysis of WBPH revealed DEGs that may contribute to the insect’s defense against the resistant rice genotypes under study. It was found that both BPH and WBPH exhibit counter-defense mechanisms in response to the various rice genes that are regulated differently during insect infestation. Gene ontology (GO) data highlighted several enriched genes in WBPH feeding on resistant lines, including those involved in “protein-containing complexes”, “enzymatic complexes”, “transferase activity”, “kinase activity”, “peptidase/endopeptidase complexes”, and the “structural composition of the cuticle” (Figure 3). These genes protect insects from the host plant’s secondary metabolites or allelochemicals and aid their survival when feeding on hosts with resistance genes. Moreover, the insects feeding on resistant hosts exhibited a significant enrichment of transcripts associated with biological processes such as “cell death”, “programmed cell death”, and “apoptotic process”. It is known that intracellular proteolytic cascades generally mediate these processes [34], and previous studies have demonstrated their importance as defense mechanisms in insects against pathogenic attacks [35]. Similar mechanisms have been observed in various insects facing viral attacks, such as the fire ant *Solenopsis invicta* [36], the fall armyworm *Spodoptera frugiperda* [37,38], and the bollworm *Helicoverpa zea* [39]. Hosts adopt avoidance, resistance, and tolerance mechanisms to cope with such infections due to the high fitness costs associated with pathogen-induced apoptosis [36,40,41]. The present study observed the enrichment of these defense pathways in insects feeding on resistant hosts. This finding raises an intriguing question regarding the potential role of these complexes in the counter-defense mechanism employed by these insects when fed on hosts carrying resistance genes. It suggests these defense pathways may significantly affect the insects’ ability to counteract the host’s resistance mechanisms. Further investigations are warranted to elucidate the precise involvement of these complexes in the defense responses of the insects and their implications in the context of host–pathogen interactions.

Interestingly, regardless of the specific rice line on which WBPH or BPH was feeding, both insects exhibited a certain level of correlation in their response to the host plants. For instance, in WBPH, we observed enrichment of terms related to the transferase complex involved in transferring phosphorus-containing groups, sulfur compound binding, organellar components, and other ion binding complexes in insects feeding on both RS and SR lines. These findings indicate that there are shared molecular mechanisms and functional pathways involved in the responses of these pests to different rice lines with resistance genes. These results suggest that the plant can sense the signals associated with piercing/sucking by insects, triggering the R (resistance) genes responses and activating downstream mechanisms to enhance resistance against insect attacks. Interestingly, this response appears independent of whether the pest is BPH or WBPH. Although triggered by different insects, this response converges on common defense pathways. As a result, a similar set of genes being activated when insects fed on rice lines carrying the RS or SR resistance genes was observed, compared to when they fed on TN1, a host that lacks any resistance genes.

Several prior studies have shown that the antixenosis mechanism involved when feeding on incompatible or resistant hosts impairs several aspects of the developmental and physiological processes of pests [42,43]. A crucial structural gene whose expression showed early and sustained induction in both insects feeding on resistant rice lines is the cuticle protein. The exoskeleton-forming cuticle protein provides mechanical support and helps with locomotion and related functions in insects [44]. It has also been reported that this protein forms a hydrophobic boundary on the insects and hence plays a role in protecting the insect from desiccation. Due to its unique permeability properties, it creates a barrier against the penetration of topically applied pesticides [45,46]. A plethora of physiological processes has been reported to be affected by the knockdown of cuticle protein, including locomotion, mechanical support, sensory perception, molting, and resistance response [44]. The molting process also involves a principal hormone in the insects, i.e., the juvenile hormone (JH) binding protein [47]. The transcriptome sequencing data indicated that in WBPH, there is a >6.0-fold upregulation of this hormone, clearly indicating that resistant hosts trigger changes in the developmental processes in these insects, specifically those related to molting and metamorphosis.

Previous studies have reported that rice leaf sheath extracts of resistant rice are highly toxic to BPH [48,49]. Also, upon BPH infestation, the rice plant produces higher levels of defense-related phenolic compounds directly correlated with the host plant resistance [49]. These phytochemicals in rice plants can influence food selection, impair feeding behavior, and consequently be detrimental to the cellular processes of these insects [50]. Among the several enzymes present in insects that are capable of inactivating plant toxins, products of the cytochrome P450 genes are vital. These mediate the hydroxylation and epoxidation required for the efficient degradation and subsequent elimination of toxins from within the insects, thereby preventing their adsorption [49,51]. Cytochrome P450s are among the primary detoxification enzymes employed by insects to cope with toxic plant compounds and help combat the plant’s chemical defenses [4]. Several studies have shown reduced tolerance to resistant hosts when genes of the cytochrome P450 family were silenced, thereby affecting the survivability of the insects [44,49,52]. In the current study, BPH showed a significantly higher induction of one of the cytochrome P450 genes when feeding on BPH-resistant hosts. In contrast, in WBPH, the expression level of another member of the P450 family of genes was not highly upregulated but showed an expression pattern similar to that observed in BPH (i.e., upregulation while feeding on the resistant host) (Figure 5 and Figure 6).

In general, as a response to infestation by BPH, rice plants that harbor resistance genes positively regulate pathways involving phytohormone signaling that, in turn, trigger a series of downstream defensive reactions, including the release of volatile compounds to repel BPH and attract its natural enemies [14,15,53,54,55]. Additionally, as a consequence, several detoxifying enzymes of the insect come into play, and these are considered significant components that the insect utilizes to cope with xenobiotics [4] present in the resistant hosts. Carboxylesterase is an essential enzyme in the insect body that detoxifies alkaloids and ferulic acid-based compounds ingested from rice [4,55]. Our study shows high induction of this gene, as observed from the initial stages of the BPH feeding on the resistant hosts. However, in contrast, in BPH feeding on susceptible hosts, only basal level expression was observed, thereby implying a potential role of this gene in contributing to the insect defense mechanism.

Similarly, differential expression levels were observed for the expression of superoxide dismutase (SOD) and peptide methionine sulfoxide reductase (PMSR) genes which are the major ROS scavenging enzymes in insects [56,57]. SODs are mainly the metalloenzymes that constitute the primary line of antioxidative defense by catalyzing the dismutation reaction and conversion of the superoxide anion radical to hydrogen peroxide and oxygen [58]. Positively induced expressions of these enzymes, as seen in the insects feeding on the resistant host, imply how the resistant genotypes are likely responsible for the accumulation of ROS required for redox signaling in the insect body [59]. ROS and other intermediates cause damage to several cellular components, and among the various amino acids, methionine is the most easily oxidized amino acid converted to methionine sulfoxide. PMSR catalyzes the reduction of methionine sulfoxide in proteins back to methionine. There is growing evidence that PMSR is critical in protecting cells against oxidative damage [56]. However, in BPH, Huang et al. [60] reported reduced expression levels when insects were transferred from a compatible host to an incompatible host. Even in the current study, we observed similar results when the insects were fed on the resistant lines. Hence, we speculate that the resistant plants induce gene suppression to ensure antibiosis.

Another vital element in the detoxification process is the ABC transporters responsible for the clearance of ingested xenobiotics in insects. In *Lygus herperus* and *Chrysomela* sp, these transporters sequester phytochemicals that form a part of the host’s arsenal of antifeedants and are ingested by these insects during feeding [61,62,63]. In this study, in WBPH, we observed significant upregulation of this gene in insects feeding on resistant genotypes and almost negligible expression when feeding on susceptible hosts. However, in BPH, an earlier study [60] reported contrasting results, where expression of this gene was relatively downregulated when transferred from a preferred host (rice) to a non-preferred host (wheat). Nonetheless, these results reveal distinct counter-defense mechanisms of these insects during host adaptation.

Sugar transporters act as osmoregulators in the insect, controlling the concentration and osmotic pressure of the plant’s phloem sap. There is a difference in the osmotic pressure in the sap-sucking insect (lower) and the plant phloem sap (higher) [63,64]. To prevent water loss in the insect body in response to the difference in osmotic pressures, osmoregulatory mechanisms have evolved in insects [63,64]. These transporters also mediate the hydrolysis of sucrose present in the ingested phloem sap to glucose and fructose before being transported across the gut epithelium. In the current study, the sugar transporter gene was upregulated in both BPH and WBPH that fed on the resistant genotypes irrespective of its specificity, i.e., in BPH, it was upregulated in insects feeding on the SR (susceptible to BPH and resistant to WBPH) line apart from the RS and RR lines, and in WBPH it was upregulated in insects feeding on the RS (resistant to BPH and susceptible to WBPH) line apart from the SR and RR lines. Therefore, the observed results suggest that any resistance gene in these RILs (RR, RS, SR) triggers the upregulation of the sugar transporter gene.

A salivary protein that helps insects survive and adapt to resistant hosts is a mucin-like protein, an essential glycoprotein found in both gelling and watery saliva, and which is specifically expressed in the salivary glands at all developmental stages of BPH [65]. In earlier studies, it has been reported that knocking down the expression of the gene coding for this protein resulted in the secretion of short and single-branched salivary sheaths, and as a result, the mucin-like deficient BPH showed disordered developmental duration, and a few insects reared on resistant rice exhibited lethality [65,66,67]. In the current study, similar differential expression responses were observed in the BPH insect fed on the resistant genotypes, thereby confirming earlier studies.

Serine proteases have been reported to play a critical role in several insects, such as the Hessian fly (*Mayetiola destructor*) and the Asian rice gall-midge (*Orseolia oryzae*) [68,69]. Studies have shown that the insect salivary gland secretions are rich in digestive enzymes, increasing the cell membrane permeability of the host while aiding insect feeding and subsequent breakdown of the ingested plant sap [69]. However, proteinase inhibitors (PIs) present in the host plants inhibit the proteases of invading insect pests leading to the starvation of the insects due to their inability to digest the plant proteins and ultimately resulting in developmental delay, reduced fecundity, and insect mortality [70]. As a part of their counter-defense, the insects tend to overproduce these enzymes to maintain normal levels of gut proteolysis, thereby facilitating the digestion of the host proteins and subsequent nutrient absorption. In the current study, it was observed that serine protease gene expression levels in BPH were higher when feeding on the resistant hosts.

The expression profile of a serine protease homolog in WBPH, i.e., the serine protease *Stubble* gene, showed upregulation in the insects feeding on the resistant hosts. This is likely due to a compensatory response aiding WBPH survival while feeding on resistant hosts. Unlike BPH, WBPH is an oligophagous insect that switches to different hosts. Therefore, it is likely that an additional serine protease homolog becomes active in WBPH, thereby allowing them to thrive on other hosts that produce different protease inhibitors (PIs) [70]. Carboxypeptidase, another essential digestive enzyme in the insects known to resist plant PIs as reported in *Helicoverpa armigera* [71], was also observed to be substantially upregulated in WBPH feeding on resistant hosts. It can be hypothesized that in WBPH, these enzymes play a critical role in counteracting the plant defense mechanism while aiding insect survival and adaptability.

An ATP-dependent RNA-helicase was found to be significantly downregulated in WBPH feeding on resistant genotypes. This gene has been reported to play an essential role in RNA metabolism [72]. Therefore, a more detailed analysis of the function of this gene in the resistant rice line could reveal molecular insights into the relationship between the expression levels of this gene in insects feeding on the resistant host genotypes and whether it subsequently affects its feeding behavior. Serine/arginine-rich splicing factors significantly regulate alternative splicing processes in eukaryotes. Alternative splicing is an important process that is technically a deviation from constitutive splicing, and contributes to proteomic expansion and alters protein functionality [73]. Knockdown of this gene in a study [44] revealed a significant decrease in the survival rates of BPH on the resistant host Mudgo, implying its critical role in insect defense mechanisms. Supporting these observations, results from the current study also show significant induction of this gene in BPH feeding on resistant hosts.

Plant cell walls, mainly composed of a pectin-embedded network of hemicellulose and cellulose [74,75], act as the main physical defenses against herbivores and pathogens by increasing the mechanical strength of plant tissues in addition to reducing the digestibility of the food consumed by the herbivores [76]. Hence, cell walls are considered the first layer of defense in plants. Insects secrete salivary cell wall degrading enzymes such as endoglucanases as a feedback mechanism to overcome such plant defenses [74,75,77]. A BPH gene, *NlEG1*, which encodes for endo-1,4-β-glucanase, was upregulated in the insects feeding on the resistant genotypes in this study. Taken together, it is possible that *NlEG1* helps BPH to feed on resistant hosts.

Insects are known to circumvent plant defenses by evolving and adopting new foraging strategies. Moreover, insect resistance is a trait that is very difficult to quantify as the resistance scores vary with the conditions of both the insects and the plant and the surrounding environment in which the experiments are being conducted [78]. Under such circumstances, using rice RILs has several advantages, as they can be easily propagated, show high reproducibility, and can be used indefinitely without additional genotyping and phenotyping. Further, the genetic background of most RILs is identical except for the trait of interest. This makes RIL populations an ideal choice for studying insect resistance. Numerous WBPH and BPH resistance genes have been identified in rice [1,13,14,15,16]. Furthermore, another interesting feature is that several resistance genes against different planthoppers may share the same loci. Based on this phenomenon, different studies have demonstrated that plants achieve a broad range of resistance against other insect species [78,79,80,81], thereby making insect–plant interaction studies a complex process. However, identifying expression (molecular) markers linked to vital traits or genes contributing to host recognition and defense might provide excellent tools to elucidate the resistance mechanisms through the metabolic pathway and network analyses. Subsequently, results from such studies could be used for developing rice improvement strategies to achieve durable resistance against different planthopper species.

The current study is the first report emphasizing the expression of genes in WBPH and BPH in response to feeding on differentially resistant RILs (F14) obtained from a cross between TN1 × RP2068. The use of different RILs with highly similar genetic backgrounds but with differential resistance to WBPH and BPH allowed us to compare the gene expression of select WBPH and BPH genes with respect to their feeding on the susceptible and resistant hosts and demonstrated the differential dynamics of the WBPH and BPH transcripts while interacting with hosts with different levels of resistance (RR, SS, SR, and RS hosts). Therefore, the observed differences in gene expression in BPH and WBPH genes are likely a result of differences in resistance traits in the lines used rather than due to differences in the genetic makeup of the hosts. The information obtained through this study is vital for utilizing natural host–plant resistance, considered one of the most economical and effective means of managing major pests, including BPH and WBPH [15]. In addition, this study enables a more profound exploration of the selective breeding and sustainable control strategies that can be adopted for developing durable resistance against these pests.

Though BPH and WBPH are considered sympatric species [7,8], under field conditions, WBPH infestation usually precedes that by BPH with a period of overlap in which rice plants are co-infested by both WBPH and BPH. The current study provides results when WPBH or BPH infests rice lines individually. Therefore, future studies designed to observe responses in the hosts as well as the pests to simultaneous infestation by both insect pests to yield valuable molecular information are warranted. Moreover, such studies are essential to confirm whether cooperative herbivory exists between WBPH and BPH, as observed in the case of double infestation of rice by BPH and striped stem borer (SSB) [82]. This study showed that BPH and SSB, with a long sympatric history together, resulted in a collaboration to overcome the rice host’s direct and indirect defense responses, and this combination benefits both herbivores. Likewise, should cooperation between WBPH and BPH exist, then similar studies are merited, for results from such investigations are likely to identify vulnerabilities in these two pests. Subsequently, we can exploit such vulnerabilities to understand better the evolution of molecular mechanisms that impact insect–plant interactions and use the information to control these pests effectively. 

The results of this study, when considered in the context of the previous study [1] relating to expression profiles of the defense genes in the corresponding RILs following infestation with either BPH or WBPH, present a holistic picture of the mutual interactions between the planthoppers and rice. Resistant RILs were shown to mount a full-fledged induced defense against BPH aiming at antibiosis while being tolerant against WBPH with increased photosynthetic activity to compensate for pest-induced nutrient loss. This is reflected in the high-level induction of the cytochrome P450 gene in BPH when feeding on the resistant RILs, and, in contrast, only a moderate response was observed of this family of genes in WBPH when feeding on its respective resistant RILs. Taken together, these results, while augmenting our understanding and knowledge of the molecular mechanisms involved in the rice–BPH/WBPH interactions, will also provide valuable information for developing lasting strategies to control these significant pests.

## 4. Materials and Methods

### 4.1. Insect Material

Laboratory-cultured BPH and WBPH were used in this study. These insects were reared on TN1 for over 45 generations at the Agri Biotech Foundation (ABF), Hyderabad (17°19′ N 78°24′ E), at 28 ± 2 °C and 70 ± 5% relative humidity under a 14/10 h light/dark photoperiod. The newly emerged female insects were transferred onto fifteen-day-old TR24SS, TR94RR, TR145RS, and TR152SR rice lines along with the TN1 rice variety and grown in separate cages. These rice RILs (F_14_) were derived from a cross between the resistant (to both BPH and WBPH) line RP2068-18-3-5 and TN1 (susceptible to both insects). SS and RR lines are, respectively, susceptible and resistant to both BPH and WBPH; RS is resistant to BPH and susceptible to WBPH, while SR is susceptible to BPH and resistant to WBPH (see Supplementary Document 1 for more details). Insect samples were collected 6 h and 12 h after infestation (hai). The collected insects were stored in TRIzol (Invitrogen, Carlsbad, CA, USA) and kept at −80 °C till further use. All experiments were carried out in triplicate (Table S1).

### 4.2. RNA Extraction 

Total RNA was extracted from BPH and WBPH insects using the RNeasy Plus Micro kit (Qiagen, Hilden, Germany) following the manufacturer’s protocol. RNA was isolated from three individual insects per group (Table S1) and later pooled as required. The integrity and quality (degradation and DNA contamination) of the isolated RNA were examined on a 0.8% agarose-formaldehyde gel [83]. The RNA was quantified using the NanoDrop Spectrophotometer (Thermo Fisher Scientific, Waltham, MA, USA). 

### 4.3. cDNA Library Preparation, Clustering, and RNA-Sequencing

RNA isolated from WBPH samples fed on TN1, TR145RS, and TR152SR was used for RNA-Seq analysis. The RNA samples collected for each of the two time points (6 hai and 12 hai) for each replication were pooled prior to sequencing by M/S Novogene (Cambridgeshire, UK). Three replicates per group were sequenced. Before proceeding with the subsequent steps, the integrity of the isolated RNA was assessed using a Bioanalyzer 2100 system (Agilent Technologies, Santa Clara, CA, USA) along with the RNA Nano 6000 Assay kit (Agilent Technologies, Santa Clara, CA, USA). One μg of total RNA per sample was used for mRNA isolation, fragmentation, and priming and for generating sequencing libraries using a NEBNext Ultra RNA Library Prep Kit for Illumina (NEB, Ipswich, MA, USA), following the manufacturer’s protocols. Index codes were added to attribute sequences to each sample. mRNA was purified from the total RNA using poly-T oligo-attached magnetic beads. First-strand cDNA was synthesized using a random hexamer primer and M-MuLV Reverse Transcriptase (RNase H), followed by second-strand cDNA synthesis using DNA Polymerase I and Rnase H utilizing the manufacturer’s protocol. The remaining overhangs were converted into blunt ends via exonuclease/polymerase activities. After adenylation of the 3′ ends of DNA fragments, NEBNext Adaptors with a hairpin loop structure were ligated to prepare for hybridization. In order to select cDNA fragments of a preferential length of 150–200 bp, the library fragments were purified with the AMPure XP system (Beckman Coulter, Beverly, MA, USA). Next, 3 μL USER Enzyme (NEB, Ipswich, MA, USA) was added to the size-selected, adaptor-ligated cDNA and incubated at 37 °C for 15 min, followed by 5 min at 95 °C before PCR. Next, PCR was performed using Phusion High-Fidelity DNA polymerase (NEB, Ipswich, MA, USA), Universal PCR primers, and Index (X) Primer as per the manufacturer’s (NEB, Ipswich, MA, USA) instructions. The PCR products were purified using the AMPure XP system, and library quality was assessed on the Agilent Bioanalyzer 2100 system using the manufacturer’s protocols. Eventually, these library preparations were sequenced on an Illumina HiSeq6000 platform, and paired-end reads were generated at M/S Novogene.

### 4.4. RNA-Seq Data Analysis 

#### 4.4.1. RNA Quality Control (QC)

The raw reads in FASTQ format were processed using fastp software (v0.20.0) [84]. The high-quality reads were processed by trimming the raw reads containing adaptor and poly-N sequences and removing reads from the raw data. GC content, Q20, and Q30 values were calculated. Further downstream analysis was carried out using high-quality read data.

#### 4.4.2. Alignment and Mapping

As a good quality reference genome for WBPH was unavailable in the database, paired-end clean reads were mapped to the reference genome of *Acyrthosiphon pisum* using HISAT-2 software (v2.0.5) [85] using the phred33 parameter. The algorithm first aligned the reads entirely to a single exon of the genome, and subsequently, the segmented reads were mapped to adjacent exons.

#### 4.4.3. Transcript Quantification 

Using default parameters, the software used to quantify transcripts generated from RNA sequencing was featureCounts (v1.5.0-p3) [86]. FPKM matrix (Fragments Per Kilobase of transcript sequence per Millions of base pairs sequenced) was used to estimate gene expression levels, considering both the sequencing depth and gene length for counting the fragments. 

#### 4.4.4. Differential Gene Expression Analysis

For each group, there were three replicates, and differential gene expression analysis was carried out using the DESeq2 R package (v1.20.0) [87]. The resulting *p* values were adjusted using Benjamini and Hochberg’s approach for controlling the False Discovery Rate (FDR) [24]. Genes with adjusted *p* values (padj ≤ 0.05) were assigned as significantly differentially expressed. Volcano plots were used to infer the overall distribution of differentially expressed genes. Cluster analysis of differentially expressed genes was performed to identify genes with similar expression patterns under various experimental conditions. Hierarchical clustering analysis was carried out using the log2 (FPKM+1) union of differentially expressed genes within the comparison of all groups. Genes within a cluster show the same trend in expression levels under different conditions. The correlation of transcripts expression between biological replicates is a critical indicator of the reliability of the experimental results. In the present study, the Pearson correlation coefficient (R^2^) was calculated based on the normalized FPKM values to reflect the transcript expression correlation between samples.

#### 4.4.5. Enrichment Analysis

The ClusterProfiler (v3.8.1) [88,89] software in the R package was used for functional enrichment analysis, including GO (Gene Ontology) and KEGG (Kyoto Encyclopedia of Genes and Genomes) enrichment.

##### Gene Ontology (GO)

GO annotated genes to biological processes, cellular components, and molecular functions, and the data were represented in a directed acyclic graph structure. GO terms with padj < 0.05 were significantly enriched by differentially expressed genes.

##### KEGG Enrichment Analysis

KEGG annotated genes to pathways, and the enrichment analysis identified significantly enriched metabolic pathways or signal transduction pathways associated with differentially expressed genes, comparing the whole genome background to the database. The compiled pathways were mapped to the differentially expressed genes. KEGG terms with padj < 0.05 were considered significantly enriched.

### 4.5. Expression Analysis of Differentially Expressed BPH and WBPH Genes by Quantitative PCR (qPCR)

Sequences of the identified differentially expressed genes (DEGs) (log2 fold > 2.5) of WBPH were retrieved from the Illumina transcriptome sequencing results. The gene identities were confirmed using BLASTx and BLASTn tools [90]. The BPH genes were selected from the NCBI database that has been previously reported to play a significant role in insect defense responses against resistant or incompatible hosts. Ten DEGs for each insect were shortlisted and used for further analysis (Table S2). Gene-specific qPCR primers were designed using the MacVector suite of sequence analysis programs (version 16.0.8, MacVector Inc., Cary, NC, USA) along with Primer 3.0 [91,92,93] software, and the sequences of the primers designed for each of the selected genes are listed in Supplementary Table S2. Total RNA (~1.5 μg) of both BPH and WBPH (both time points; 6 and 12 hai) was reverse transcribed to cDNA using SuperScript IV first strand cDNA synthesis system (Invitrogen, Carlsbad, CA, USA) following the manufacturer’s protocol, and it was quantified using the NanoDrop Spectrophotometer (Thermo Fisher Scientific, Waltham, MA, USA). qPCR (RT-PCR) was performed using ABI 7500 real-time PCR system using PowerUp SYBR Green master mix (Thermo Fisher Scientific, Waltham, MA, USA). Each real-time PCR reaction of 10 μL contained 5 μL SYBR Green master mix, 5 μM each of the forward and reverse primers, and 5 ng cDNA template. PCR conditions involved initial denaturation at 95 °C for 5 min, followed by 40 cycles of denaturation at 95 °C for 10 s, followed by annealing for 15 s (at primer set-specific temperatures; see Table S2), and extension at 60 °C for 30 s. The transcript abundance was estimated using *actin* as the endogenous control for both BPH and WBPH. The data were analyzed according to the 2^−ΔΔCT^ method using the geometric mean of the *actin* gene for normalization using Applied Biosystem 7500 software (v2.3) (Thermo Fisher Scientific, Waltham, MA, USA). A non-template negative control (NTC) was included for each primer set to confirm and check for the absence of contamination or primer dimers in the reactions. Each sample contained three biological replicates, and each biological replicate had three technical replicates. The data of the two time points (6 and 12 hai) were analyzed and grouped into a single study using Applied Biosystem 7500 software.

### 4.6. Data Analyses

The data analysis was carried out using GraphPad Prism 9 software (version 9.4.0; Boston, MA, USA). The results were presented as mean ± SE (standard error). One-way analysis of variance (ANOVA) followed by a Šidák multiple comparison tests was carried out to confirm the statistical significance of the gene expression differences between the groups (Table S1) under study (*p* < 0.05) [94].

## Figures and Tables

**Table 1:** QC summary of the RNA-Seq data *.

Sample Name	Raw Reads	Clean Reads	Raw Bases	Clean Bases	Error Rate (%)	Q20 (%)	Q30 (%)	GC Content (%)
W_TN1-R1	24206186	23584788	7.3G	7.1G	0.03	97.91	93.96	37.47
W_TN1-R2	21448759	21053170	6.4G	6.3G	0.03	97.89	93.86	38.65
W_TN1-R3	21926318	21522130	6.6G	6.5G	0.03	97.94	94.02	37.92
W_TR145RS_R1	26899128	26352877	8.1G	7.9G	0.03	97.95	94.01	38.2
W_TR145RS_R2	23710772	23313968	7.1G	7.0G	0.03	98	94.09	38.87
W_TR145RS_R3	25541544	25066110	7.7G	7.5G	0.02	98.08	94.28	39.75
W_TR152SR-R1	22617866	22093361	6.8G	6.6G	0.02	98.16	94.45	38.2
W_TR152SR-R2	22276222	21481497	6.7G	6.4G	0.03	97.92	93.94	38.62
W_TR152SR-R3	21430485	20979347	6.4G	6.3G	0.03	97.91	93.89	38.82

* TN1: Control (susceptible to both hoppers); TR145RS: Susceptible to WBPH (resistant to BPH); TR152SR: Resistant to WBPH (susceptible to BPH); R1, R2, R3: Three biological replicates.

## Data Availability

The sequence data generated for this study have been deposited at the NCBI SRA (Sequence Read Archive) database and can be accessed using the following BioProject accession number: PRJNA865545.

## Supplementary Materials

The supporting information can be downloaded at: https://www.mdpi.com/article/10.3390/ijms241813982/s1.
