# Supplementary material for: Expression Analysis Reveals Differentially Expressed Genes in BPH and WBPH Associated with Resistance in Rice RILs Derived from a Cross between RP2068 and TN1
Source: Int J Mol Sci. 2023 Sep 12;24(18):13982. doi: 10.3390/ijms241813982 (PMC10531025; doi:10.3390/ijms241813982)
Supplement: Supplementary file 1 [file ijms-24-13982-s001.zip › Figure S1.pdf]

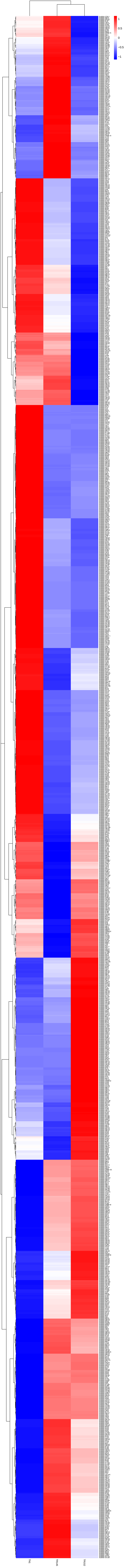
1
0.5
0
-0.5
-1
TN1
TR145
TR152

Supplementary Figure S1: Hierarchical clustering heatmap depicting overall results of the FPKM clustering of differentially expressing genes (DEGs) of WBPH (using log2 (FPKM+1) values) while feeding on different RILs (TN1, TR145 and TR152). The scaffold IDs are provided on the right. The color scale represents an increase in gene expression from blue (-1) to red (1).
