# Supplementary material for: Expression Analysis Reveals Differentially Expressed Genes in BPH and WBPH Associated with Resistance in Rice RILs Derived from a Cross between RP2068 and TN1
Source: Int J Mol Sci. 2023 Sep 12;24(18):13982. doi: 10.3390/ijms241813982 (PMC10531025; doi:10.3390/ijms241813982)
Supplement: Supplementary file 1 [file ijms-24-13982-s001.zip › Supplementary Document 1 revised.pdf]

Supplementary Materials and Methods

Details of the tests conducted for selection of the four RILs (RR, SS, RS and SR) used for this study.

According to our earlier study reported by Divya et al. [1], a total of 180 Recombinant Inbred Lines (RILs; F_{14}) derived from a cross between RP2068-18-3-5 (resistant to both BPH and WBPH) and TN1 (susceptible to both hoppers), were subjected to four phenotypic tests to evaluate their resistance against BPH and WBPH infestations. The details of these tests are as follows:

Seedbox Screen test: The RILs were screened in triplicates using a seedbox test. Each test plant was evaluated for damage based on the Standard Evaluation System (SES), and the damage score was recorded.

Nymphal Survival test: The test plants were infested with 1st or 2nd instar BPH and WBPH insects. Three replicates were maintained for this test. The number of surviving insects was observed and recorded daily until all the surviving nymphs metamorphosed into adults. Nymphal survival was expressed as a percentage. RILs supporting more than 60% BPH or WBPH nymphal survival rate were considered susceptible.

Days to Wilt test: This test measured the tolerance component of resistance. The RILs were potted, and 50 BPH/WBPH insects were released separately. The experiment was conducted in replicates. The plants were monitored daily, and the day of complete wilting was recorded. Plants that survived for more than 10 days were considered resistant, while those that wilted earlier were considered susceptible.

Nymphal preference test: This test aimed to observe antixenosis or non-preference. The number of nymphs on each seedling was recorded after 24 and 48 hours of infestation. The percentage of insects settled on each test plant was calculated based on the observed number of insects for each replication.

All the data obtained from these phenotypic tests were subjected to statistical analysis using One-way ANOVA. The means were separated using the HSD (Honestly Significant Difference) test following the Tukey and Kramer method.

Additionally, molecular markers were used to analyse the selected RILs further. 137 polymorphic molecular markers were screened on the RILs (shortlisted based on the phenotypic tests). The marker-based screening revealed that the RILs possessed genetic similarity ranging from 44% to 84%. Based on the results of these tests, the study concluded that the genes conferring resistance to BPH and WBPH were independent of each other. Further, the shortlisted RILs were classified as RR, SS, RS and SR and were used in this study.
